# Targeting cytomegalovirus (CMV) antigens for GBM immunotherapy

**DOI:** 10.1186/2051-1426-1-S1-P270

**Published:** 2013-11-07

**Authors:** Smita K Nair, Gabriel De Leon, David Boczkowski, Robert Schmittling, Weihua Xie, Gerald Archer, John H Sampson, Duane A Mitchell

**Affiliations:** 1 Surgery, Duke University Medical Center, Durham, NC, USA; 2 Neurosurgery, University of Florida, Gainesville, FL, USA

## 

Despite aggressive and incapacitating conventional therapy, glioblastoma multiforme (GBM) remains uniformly lethal. Immunotherapy in which the immune system (particularly cellular effectors such as T cells) is harnessed to specifically attack malignant cells offers a treatment option with less toxicity. The presence of CMV antigens in GBM, and not in normal brain tissue presents a unique opportunity to target these viral proteins for tumor immunotherapy. Although many labs, including ours, have demonstrated the presence of CMV proteins in GBM, their relevance as immunological targets in GBM has yet to be established. The hypothesis of this study is that CMV proteins in GBM are relevant tumor antigens based on their immunogenicity and restricted expression within GBM, and immunological targeting of CMV will result in an anti-GBM immune response. The objective of this study is to demonstrate that CMV pp65-specific T cells target and eliminate GBM tumor cells in an antigen-specific manner. To achieve this objective, we first demonstrate the ability to generate CMV pp65-specific immune responses in vitro using peripheral blood mononuclear cells (PBMCs) from GBM patients, indicated by the increase in CMV-specific tetramer positive cells and IFN-gamma production. Primary GBM specimens were shown to express early and late CMV gene products (IE1, pp65, gB) by Western blot analysis. Importantly, CMV pp65-specific T cells generated by in vitro stimulation of patient T cells with dendritic cells transfected with CMV pp65 mRNA specifically lyse autologous, primary GBM tumor cells. Moreover, tumor-specific T cells, generated by in vitro stimulation with dendritic cells transfected with total tumor RNA derived from GBM specimens, stimulate CMV pp65-specific T cells as evidenced by an increase in CMV pp65-specific tetramer positive cells. These data collectively demonstrate that CMV proteins can be targeted for GBM immunotherapy and CMV antigen-specific responses are effective at recognizing and lysing autologous GBM tumor cells.

